# Dose-response relationship between physical activity and visceral fat mass: a cross-sectional study based on NHANES 2011–2018

**DOI:** 10.1186/s12889-025-24393-6

**Published:** 2025-09-24

**Authors:** Jiancheng Bo, Mingyue Yang

**Affiliations:** 1 Department of Sports Medicine, The First Affiliated Hospital of Xinxiang Medical University, Xinxiang, China; 2 Department of Anesthesiology, The First Affiliated Hospital of Xinxiang Medical University, Xinxiang, China

**Keywords:** Physical activity, Visceral fat mass, NHANES, Cross-Sectional study, Adults, Dose-Response relationship

## Abstract

Excess visceral fat mass (VFM) is a key risk factor for metabolic diseases, and physical activity (PA) is a modifiable behavior associated with lower VFM. This cross-sectional study investigates the dose-response relationship between PA and VFM, with a focus on differences by sex and race/ethnicity, using data from the National Health and Nutrition Examination Survey (NHANES) 2011–2018. Among 9,111 adults aged 20–59 years, PA was measured as metabolic equivalent of task (MET) minutes per week via the Global Physical Activity Questionnaire (GPAQ) and categorized into quintiles (Q1: 0-280, Q2: 281-1,680, Q3: 1,681-5,400, Q4: 5,401 − 11,020, Q5: >11,020 MET-min/week). VFM was assessed using dual-energy X-ray absorptiometry (DXA). Weighted linear regression and restricted cubic spline (RCS) models were employed to examine associations, adjusting for demographic (age, sex, race/ethnicity), lifestyle (smoking, alcohol, caloric intake), and health-related (BMI, diabetes, hypertension) covariates. Higher PA levels were associated with lower VFM ratios, with the highest quintile (Q5) having a 12% lower ratio compared to the lowest (Q1) (Ratio = 0.88, 95% CI: 0.85–0.91, *P* < 0.001) after full adjustment.RCS analyses revealed sex-specific patterns: males exhibited a monotonic decrease in VFM up to 50,000 MET-min/week, while females showed a U-shaped association, with the lowest VFM at ~ 6,000 MET-min/week, suggesting moderate PA (~ 5,000–7,000 MET-min/week) as optimal. Significant interactions by sex (*P*_interaction = 0.008) and race/ethnicity (*P*_interaction < 0.001) highlighted heterogeneity: Non-Hispanic Whites showed a consistent VFM decline, while Mexican Americans exhibited a weaker association with An inverted U-shape, peaking at 1,000–3,000 MET-min/week. These findings underscore the need for tailored PA recommendations by sex and race/ethnicity. The cross-sectional design precludes causal inference.

**Supplementary Information:**

The online version contains supplementary material available at 10.1186/s12889-025-24393-6.

## Introduction

Visceral fat, an adipose tissue surrounding internal organs within the abdominal cavity, is metabolically distinct from subcutaneous fat due to its higher lipolytic activity and capacity to secrete pro-inflammatory cytokines and adipokines [1, 2]. Its accumulation is strongly Linked to increased risks of cardiovascular disease, type 2 diabetes, and metabolic syndrome [3–7]. While calorie restriction can be associated with lower visceral fat mass (VFM), it is prone to rebound effects and may impact overall health [8, 9]. In contrast, physical activity (PA) promotes metabolic health, reduces risks of chronic diseases, and serves as a key preventive measure [10, 11].

There have been several studies examining the relationship between physical activity and visceral fat. Rottensteiner et al. selected 10 pairs of young adult male identical twins and found that lower levels of physical activity were associated with greater accumulation of intra-abdominal fat [12]. Waters et al., in an 18-month randomized controlled trial, reported that aerobic exercise independently contributed to lower VFM [13]. Similarly, Murabito et al., using accelerometry, observed that moderate-to-vigorous PA was associated with lower visceral and subcutaneous adipose tissue and improved fat quality [14].

Despite these findings, limitations in the literature underscore the need for further research. Many studies rely on small or homogenous samples, limiting generalizability [12, 14, 15]. PA assessment methods vary, with self-reported data prone to recall bias and objective measures like accelerometry yielding inconsistent results [14, 16, 17]. Notably, few studies have examined how the PA-VFM association varies by demographic factors such as sex and race/ethnicity, which are known to influence fat distribution and metabolic responses to PA [18–20]. For example, women may exhibit non-linear responses to PA due to hormonal influences, and racial/ethnic groups may differ in VFM accumulation patterns [19, 20].

The National Health and Nutrition Examination Survey (NHANES) provides a robust platform to address these gaps, utilizing a complex, stratified sampling design that ensures national representativeness of the U.S. non-institutionalized population. While NHANES has been widely used to study obesity and metabolic health [21, 22]few studies have specifically investigated the dose-response relationship between PA and VFM, particularly with a focus on non-linear patterns and subgroup differences by sex and race/ethnicity[23, 24]. This study aims to examine the dose-response association between PA And VFM in 9,111 adults aged 20–59 years from NHANES 2011–2018, with a particular emphasis on differences by sex and race/ethnicity. We hypothesize that higher PA levels are associated with lower VFM, with non-linear patterns and variations across sex and race/ethnicity, potentially due to differences in hormonal regulation and fat distribution.

## Methods

### Study design and population

**Fig. 1:**
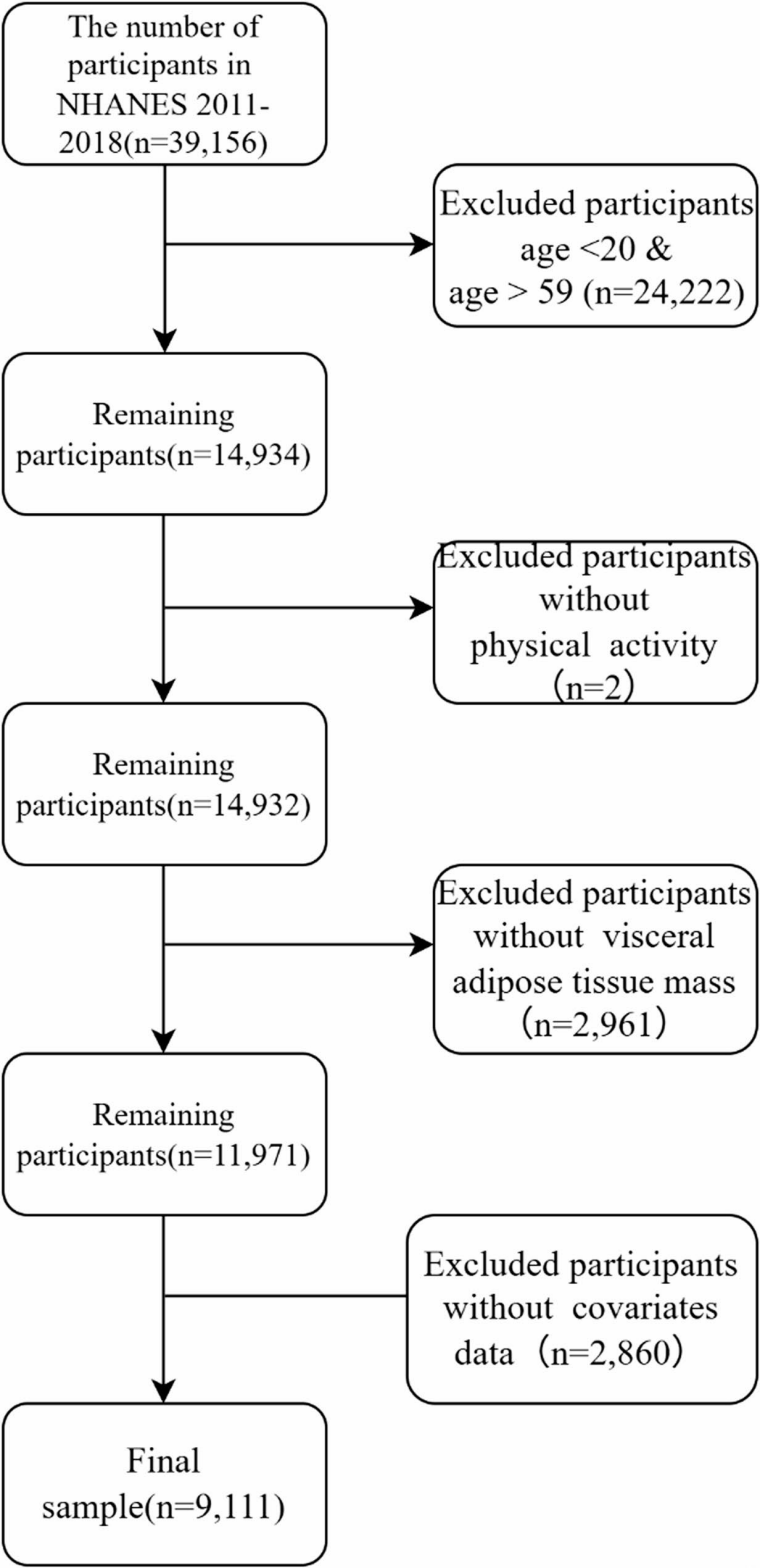
Flow chart for participants

This cross-sectional study utilized data from the National Health and Nutrition Examination Survey (NHANES) 2011–2018, encompassing 39,156 participants across four cycles. NHANES employs a stratified, multistage probability sampling design to represent the U.S. non-institutionalized population [25]. The NHANES protocol was approved by NCHS Ethics Review Board, with written informed consent obtained from all participants. From 39,156 participants across four cycles, we included adults aged 20–59 years who underwent DXA scans and completed the Physical Activity Questionnaire (PAQ). This age restriction was applied because: (1) DXA scans are only available for ages 8–59 years in NHANES, (2) it minimizes metabolic differences between adolescents and adults, and (3) reduces confounding from age-related changes (e.g., sarcopenia) in older adults [26]. Exclusion criteria included: age < 20 & >59 years (*n* = 24,222 excluded), pregnancy, missing PA or visceral fat mass (VFM) data (*n* = 2,963), and missing covariates (e.g., education, BMI, diabetes, hypertension, smoking, alcohol use, caloric intake; additional exclusions) (*n* = 2,860). The final Analytic sample consisted of 9,111 participants (see Fig. 1).

### Measurement of visceral fat mass

Visceral fat mass (VFM) was measured using dual-energy X-ray absorptiometry (DXA) with a Hologic Discovery A scanner (Hologic Inc., Bedford, MA) in the NHANES mobile examination centers. This method provides a low-radiation, high-precision assessment of body composition [27, 28]. The scans were analyzed using Hologic APEX software (version 4.0), which employs a validated algorithm to estimate VFM within the android region, specifically approximating the adipose tissue volume at the L4-L5 vertebral interspace [29]. This DXA-based VFM estimation has been shown to be highly correlated with gold-standard measurements from computed tomography (CT) and magnetic resonance imaging (MRI) [30]. Participants were excluded from the analysis if they exceeded the DXA table limits (weight > 450 lbs or height > 6’5”) or had used radiopaque contrast agents recently. All data collection and quality control procedures adhered to the standardized NHANES protocols.

### Physical activity assessment

PA was assessed via the PAQ, adapted from the Global Physical Activity Questionnaire (GPAQ), capturing work, leisure, and transportation-related activities during a typical week, reflecting usual activity patterns. MET minutes per week were calculated as: Total MET = (8 × vigorous work) + (4 × moderate work) + (8 × vigorous leisure) + (4 × moderate leisure) + (4 × transportation), per NHANES guidelines [31, 32]. Participants were categorized into five MET-based activity groups (quintiles): Q1 (0-280), Q2 (281-1,680), Q3 (1,681-5,400), Q4 (5,401 − 11,020), Q5 (11,021–59,040) MET-min/week[33]. This quintile approach was selected to capture the full spectrum of PA levels within the NHANES 2011–2018 population, reflecting the right-skewed distribution of MET values where higher PA levels (Q4 and Q5, totaling ~ 25%) are less prevalent due to the inclusion of both recreational and occupational activities [33]. MET-minutes per week was preferred over minutes/week as it accounts for activity intensity and duration, enabling a more precise dose-response analysis, and aligns with NHANES standardized methodology for assessing energy expenditure [31].The data-driven quintile division ensures representation across the range and facilitates detection of non-linear dose-response relationships, as validated by restricted cubic spline analyses.

### Covariates

Covariates were selected based on their established associations with visceral fat mass (VFM) and physical activity (PA) in prior literature, ensuring comprehensive adjustment for potential confounders [24, 34]. These included age (continuous, in years; categorized as young adults [20–35 years], middle-aged [36–45 years], older adults [46–59 years] for subgroup analyses), sex (male, female), race/ethnicity (Mexican American, Other Hispanic, Non-Hispanic White, Non-Hispanic Black, Other), marital status (never married, widowed/divorced, married/living with partner), education level (below high school, high school, above high school), smoking status (never, former, current), alcohol consumption (no, yes), average daily caloric intake (derived from two 24-hour dietary recalls using the USDA’s Automated Multiple-Pass Method) [35]body mass index (BMI, kg/m²; categorized as normal [< 25], overweight [25-29.9], obesity [≥ 30] for descriptive purposes), diabetes (defined by self-report of physician diagnosis, fasting glucose ≥ 126 mg/dL, or HbA1c ≥ 6.5%[36]), hypertension (based on self-reported physician diagnosis [37]). Data were collected through standardized NHANES interviews, physical examinations, and laboratory assessments conducted in mobile examination centers, with detailed protocols available in the NHANES Analytic Guidelines [25]. Self-reported hypertension data were used, supported by NHANES validation studies showing high agreement with clinical measures (κ = 0.75)[38].This approach balanced data completeness (minimizing exclusions) and reliability, with potential misclassification addressed by adjusting for metabolic confounders (BMI, diabetes).

### Statistical analysis

All analyses were conducted using R software (version 4.4.2) with the survey package to account for NHANES’ complex sampling design, alongside the rms package for restricted cubic spline (RCS) modeling. Due to the right-skewed distribution of VFM (skewness = 0.97), VFM was log-transformed prior to analysis. For descriptive statistics, continuous variables were summarized as means ± standard deviations (SD) if normally distributed, or as medians with interquartile ranges (IQR) if non-normally distributed. Categorical variables were reported as unweighted sample sizes (n) with weighted percentages (%), and differences across groups were tested using design-adjusted Kruskal-Wallis tests for continuous variables or Rao-Scott chi-square tests for categorical variables (*P* < 0.05).

To evaluate the association between MET and VFM, two weighted linear regression models were fitted using the svyglm function from the survey package in R, which accounts for the complex survey design of NHANES: Model 1 was unadjusted; Model 2 adjusted for age, sex, race/ethnicity, marital status, education level, smoking, alcohol consumption, daily caloric intake, BMI, diabetes, and hypertension. Trend tests were performed by treating MET quintiles as a continuous variable. The regression coefficients were exponentiated to obtain ratios (exp(*β*)), representing the relative change in VFM compared to the reference group (Q1). Model assumptions, including normality of residuals and homoscedasticity, were verified using residual plots and Q-Q plots.

Non-linear associations were explored using RCS models with four knots placed at the 5th, 35th, 65th, And 95th percentiles of log-transformed total MET (log_total_met), stratified by sex, among participants with total MET ≥ 40 min/week. Subgroup analyses were conducted by sex, race/ethnicity, age, BMI, diabetes, and hypertension, with interaction effects assessed using interaction tests (*P*_interaction < 0.05). Sensitivity analyses were performed using alternative PA categorizations (e.g., Fowler et al.’s classification: <500, 500-1,000, 1,000–1,500 MET-min/week) and models with hypercholesterolemia to assess result robustness.

### Sensitivity analyses

To evaluate the robustness of our primary findings, we conducted two sensitivity Analyses. First, we extended Model 2 by including hypercholesterolemia as an additional covariate, defined as a self-reported physician diagnosis, or measured total cholesterol ≥ 240 mg/dL, consistent with the NCEP ATP III guidelines and NHANES protocols [25, 39]. Second, we replaced the quintile-based categorization with an alternative physical activity (PA) classification proposed by Fowler et al. [40]adapted to align with the 2018 US Physical Activity Guidelines [41]. Total MET-min/week was categorized as follows: Inactive (0 MET-min/week, no activity reported), Low (40–499 MET-min/week), Moderate (500–999 MET-min/week), High (1,000–1,499 MET-min/week), and Very High (≥ 1,500 MET-min/week). This approach aimed to address potential misclassification in the primary analysis while exploring guideline-aligned PA thresholds.

## Results

### Sample characteristics

Among 9,111 participants (Table 1), the mean age was 39.47 ± 11.77 years, BMI was 29.13 ± 6.90 kg/m², And 51.67% were male. The median visceral fat mass (VFM) was 464.04 g (IQR: 286.70-684.10), decreasing across MET quintiles (Q1: 581.99 g; Q5: 419.74 g; *P* < 0.001). Of the participants, 1,770 (17.37%) reported no moderate-to-vigorous physical activity, accounting for 75.87% of the Q1 group. Participants in higher MET quintiles were younger (Q1: 42.54 ± 11.43 years; Q5: 36.52 ± 11.35 years), had slightly higher daily caloric intake (Q1: 2,074.04 ± 840.39 kcal; Q5: 2,449.41 ± 1,064.80 kcal), and included a greater proportion of males (Q1: 40.54%; Q5: 73.63%). Married or partnered individuals were slightly less likely to engage in higher levels of physical activity (Q1: 66.75%; Q5: 60.45%). Compared to those with no activity, participants with higher MET levels had lower prevalence of diabetes (Q1: 10.03%; Q5: 4.50%) and hypertension (Q1: 30.45%; Q5: 20.56%). Significant differences (*P* < 0.001) were observed across MET groups for all variables, including age, BMI, caloric intake, sex, race/ethnicity, marital status, education, smoking, and alcohol use.

**Table 1:** The characteristics of participants (NHANES 2011–2018)

Variable	Overall (*N* = 9,111)	MET-Based Activity Groups					*P*-value
		**Q1** (*N* = 2,297)	**Q2** (*N* = 2,358)	**Q3** (*N* = 2,190)	**Q4** (*N* = 1,125)	**Q5** (*N* = 1,141)	
Visceral fat mass (g)	464.04 (286.70, 684.10)	581.99 (375.30, 804.53)	461.27 (292.32, 678.47)	412.50 (259.03, 614.83)	420.73 (266.92, 634.00)	419.74 (259.60, 622.30)	< 0.001
Age (years)	39.47 ± 11.77	42.54 ± 11.43	40.16 ± 11.37	38.70 ± 11.83	36.90 ± 12.01	36.52 ± 11.35	< 0.001
BMI (kg/m2)	29.13 ± 6.90	30.95 ± 7.56	28.82 ± 6.68	28.25 ± 6.44	28.81 ± 6.70	28.57 ± 6.58	< 0.001
Energy intake (kcal/day)	2,190.82 ± 880.03	2,074.04 ± 840.39	2,107.62 ± 772.76	2,180.67 ± 825.46	2,341.45 ± 988.55	2,449.41 ± 1,064.80	< 0.001
Physical activity level (MET-min/week)	1,800 (360, 5,520)	0.00 (0, 0)	960 (600, 1,360)	2,880 (2,280, 3,880)	7,440 (6,336, 9,120)	16,800 (13,440, 22,080)	< 0.001
Zero activity proportion (%)							< 0.001
Active	7,341 (82.63%)	527 (24.13%)	2,358 (100.00%)	2,190 (100.00%)	1,125 (100.00%)	1,141 (100.00%)	
Inactive	1,770 (17.37%)	1,770 (75.87%)	0 (0.00%)	0 (0.00%)	0 (0.00%)	0 (0.00%)	
Sex, n (%)							< 0.001
Male	4,666 (51.67%)	920 (40.54%)	1,032 (43.81%)	1,208 (54.12%)	680 (61.59%)	826 (73.63%)	
Female	4,445 (48.33%)	1,377 (59.46%)	1,326 (56.19%)	982 (45.88%)	445 (38.41%)	315 (26.37%)	
Age group, n (%)							< 0.001
Young adults (20–35 years)	3,775 (40.35%)	703 (28.68%)	919 (37.84%)	1,028 (43.91%)	563 (49.11%)	562 (50.92%)	
Middle-aged (36–45 years)	3,098 (36.24%)	1,015 (46.83%)	841 (38.52%)	638 (33.58%)	308 (27.87%)	296 (25.93%)	
Older adults (46–59 years)	2,238 (23.41%)	579 (24.49%)	598 (23.64%)	524 (22.51%)	254 (23.02%)	283 (23.14%)	
Race/ethnicity, n (%)							< 0.001
Mexican American	1,290 (9.73%)	369 (11.56%)	281 (7.80%)	282 (8.48%)	171 (10.58%)	187 (12.16%)	
Other Hispanic	938 (6.91%)	278 (8.41%)	205 (5.63%)	234 (6.99%)	101 (6.30%)	120 (7.32%)	
Non-Hispanic White	3,342 (62.97%)	718 (56.93%)	870 (65.40%)	837 (65.76%)	449 (64.10%)	468 (62.03%)	
Non-Hispanic Black	2,021 (11.59%)	525 (12.98%)	505 (10.97%)	435 (9.62%)	279 (12.78%)	277 (13.19%)	
Other Race	1,520 (8.80%)	407 (10.13%)	497 (10.19%)	402 (9.15%)	125 (6.24%)	89 (5.30%)	
Marital status, n (%)							< 0.001
Never married	2,460 (24.61%)	496 (19.55%)	600 (21.68%)	685 (27.47%)	360 (31.39%)	319 (27.41%)	
Widowed/divorced	1,183 (12.15%)	341 (13.70%)	310 (11.91%)	248 (11.39%)	141 (11.37%)	143 (12.14%)	
Married/living with partner	5,468 (63.24%)	1,460 (66.75%)	1,448 (66.41%)	1,257 (61.14%)	624 (57.25%)	679 (60.45%)	
Education, n (%)							< 0.001
Below high school	1,609 (12.84%)	538 (17.30%)	323 (9.56%)	305 (9.73%)	200 (14.10%)	243 (16.74%)	
High school	5,504 (65.50%)	1,241 (59.46%)	1,611 (73.04%)	1,482 (73.46%)	654 (62.37%)	516 (47.50%)	
Above high school	1,998 (21.66%)	518 (23.25%)	424 (17.40%)	403 (16.81%)	271 (23.53%)	382 (35.76%)	
BMI Category, n (%)							< 0.001
Normal (< 25)	2,760 (30.07%)	590 (22.17%)	757 (30.99%)	726 (33.82%)	336 (32.29%)	351 (32.68%)	
Overweight (25 ~ 29.9)	2,838 (31.69%)	654 (27.81%)	717 (31.27%)	749 (35.00%)	361 (32.35%)	357 (32.26%)	
Obesity (≥ 30)	3,513 (38.23%)	1,053 (50.02%)	884 (37.74%)	715 (31.18%)	428 (35.36%)	433 (35.06%)	
Smoking, n (%)							< 0.001
Never	5,484 (58.62%)	1,394 (57.93%)	1,507 (61.72%)	1,401 (62.80%)	651 (56.74%)	531 (46.68%)	
Former	1,550 (19.90%)	371 (18.63%)	411 (21.58%)	360 (18.48%)	197 (20.98%)	211 (20.45%)	
Current	2,077 (21.48%)	532 (23.43%)	440 (16.69%)	429 (18.72%)	277 (22.28%)	399 (32.86%)	
Alcohol intake, n (%)							< 0.001
No	2,025 (16.99%)	669 (22.46%)	569 (17.98%)	415 (15.22%)	209 (14.07%)	163 (11.43%)	
Yes	7,086 (83.01%)	1,628 (77.54%)	1,789 (82.02%)	1,775 (84.78%)	916 (85.93%)	978 (88.57%)	
Diabetes, n (%)							< 0.001
No	8,358 (93.35%)	2,032 (89.97%)	2,156 (93.11%)	2,044 (94.87%)	1,048 (94.77%)	1,078 (95.50%)	
Yes	753 (6.65%)	265 (10.03%)	202 (6.89%)	146 (5.13%)	77 (5.23%)	63 (4.50%)	
Hypertension, n (%)							< 0.001
No	6,907 (76.95%)	1,616 (69.55%)	1,788 (78.04%)	1,748 (79.51%)	879 (80.47%)	876 (79.44%)	
Yes	2,204 (23.05%)	681 (30.45%)	570 (21.96%)	442 (20.49%)	246 (19.53%)	265 (20.56%)	

Statistics are based on NHANES complex survey design for weighted estimates

*P*-values were calculated using design-based Kruskal-Wallis tests for continuous variables and Rao-Scott adjusted chi-square tests for categorical variables. Continuous variables are presented as mean ± SD unless otherwise specified; Visceral fat mass (g) and Physical activity level (MET-min/week) are presented as median [IQR]

Participants were categorized into five MET-based activity groups (MET-min/week): Q1 (0-280), Q2 (281-1,680), Q3 (1,681-5,400), Q4 (5,401 − 11,020), Q5 (11,021–59,040)

### Dose-Response relationship

Table 2 presents the association between MET and VFM. In the unadjusted model (Model 1), Q5 vs. Q1 showed a 25% reduction in VFM (Ratio = 0.75, 95% CI: 0.71–0.80, *P* < 0.001), with a decreasing trend across quintiles (Q2: 0.82; Q3: 0.74; Q4: 0.76). After full adjustment (Model 2), the reduction in Q5 vs. Q1 attenuated to 12% (Ratio = 0.88, 95% CI: 0.85–0.91, *P* < 0.001), with a consistent trend across quintiles (Q2: 0.96; Q3: 0.90; Q4: 0.91; *P*_trend < 0.001), indicating that higher MET levels were associated with lower VFM.

**Table 2:** Dose-response relationship between MET-based activity groups and visceral fat mass using weighted linear regression

Model	Q1 (0-280 MET-min/week)	Q2 (281-1,680 MET-min/week)	Q3 (1,681-5,400 MET-min/week)	Q4 (5,401 − 11,020 MET-min/week)	Q5 (11,021–59,040 MET-min/week)	Trend *P*-value
N	2,298	2,359	2,190	1,124	1,141	
Model 1	Ref	0.82 (0.78, 0.86)	0.74 (0.71, 0.78)	0.76 (0.72, 0.80)	0.75 (0.71, 0.80)	< 0.001
Model 2	Ref	0.96 (0.94, 0.99)	0.90 (0.88, 0.93)	0.91 (0.88, 0.94)	0.88 (0.85, 0.91)	< 0.001

Note: Ratios (exp(β)) represent the relative change in visceral fat mass (log-transformed) compared to Q1, estimated using weighted Linear regression. Model 1: Unadjusted; Model 2: Adjusted for age, sex, race/ethnicity, marital status, education level, smoking, alcohol consumption, daily caloric intake, BMI, diabetes, and hypertension.

Trend *P*-value was calculated by treating MET quintiles as a continuous variable. Participants were categorized into five MET-based activity groups (MET-min/week): Q1 (0-280), Q2 (281-1,680), Q3 (1,681-5,400), Q4 (5,401 − 11,020), Q5 (11,021–59,040)

### Subgroup analysis

**Fig. 2:**
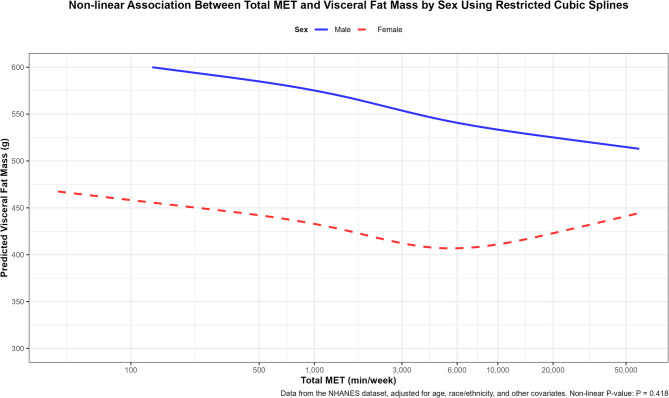
Sex-specific association between total MET and visceral fat mass using restricted cubic splines. Note: Data from the NHANES dataset (2011–2018), including participants with total MET ≥ 40 min/week, adjusted for age, race/ethnicity, marital status, education level, smoking, alcohol consumption, daily caloric intake, BMI, diabetes, And hypertension. RCS models used four knots at the 5th, 35th, 65th, And 95th percentiles of log-transformed total MET. The x-axis represents total MET (min/week), ranging from 40 to 50,000, displayed on a natural logarithmic scale (ln) with breaks at 100, 500, 1,000, 3,000, 6,000, 10,000, 20,000, And 50,000. Non-linear *P*-value: *P* = 0.418

Table 3 confirms a consistent negative association between MET and visceral fat mass (VFM) across all subgroups, with significant modifications by sex and race/ethnicity (*P*_interaction = 0.008 and < 0.001, respectively). In males, the association was monotonic, with VFM decreasing progressively across MET quintiles (Q5 vs. Q1: Ratio = 0.89, 95% CI: 0.85–0.92; Q2-Q4: 0.97, 0.91, 0.92). In females, the association was less consistent: VFM decreased modestly from Q2 to Q3 (Q2: 0.96, 95% CI: 0.93–0.99; Q3: 0.90, 95% CI: 0.86–0.94) but plateaued from Q3 to Q5 (Q4: 0.90, 95% CI: 0.86–0.95; Q5: 0.90, 95% CI: 0.83–0.97). This plateau aligns with the U-shaped pattern observed in the RCS analysis (Fig. 2), where female VFM reached a nadir (~ 410 g) at 6,000 MET-min/week before increasing to ~ 450 g at 50,000 MET-min/week, suggesting that excessive PA may not yield additional benefits and could be associated with VFM increases [42].

**Table 3:** Subgroup analysis of the dose-response relationship between MET-based activity groups and visceral fat mass using weighted linear regression

Variable	Subgroups	Q1	Q2 Ratio (95% CI)	Q3 Ratio (95% CI)	Q4 Ratio (95% CI)	Q5 Ratio (95% CI)	*P* for interaction
Sex							0.008
	Male	Ref	0.97 (0.94–1.01)	0.91 (0.88–0.93)	0.92 (0.89–0.95)	0.89 (0.85–0.92)	
	Female	Ref	0.96 (0.93–0.99)	0.90 (0.86–0.94)	0.90 (0.86–0.95)	0.90 (0.83–0.97)	
Race/ethnicity							< 0.001
	Mexican American	Ref	0.99 (0.93–1.06)	0.95 (0.89–1.02)	0.99 (0.94–1.03)	0.96 (0.90–1.02)	
	Other Hispanic	Ref	0.95 (0.89-1.00)	0.87 (0.82–0.91)	0.84 (0.77–0.92)	0.92 (0.86–0.98)	
	Non-Hispanic White	Ref	0.97 (0.94–1.01)	0.91 (0.87–0.94)	0.91 (0.87–0.95)	0.87 (0.82–0.92)	
	Non-Hispanic Black	Ref	0.95 (0.90-1.00)	0.92 (0.87–0.97)	0.95 (0.89–1.01)	0.93 (0.87–0.99)	
	Other	Ref	0.96 (0.91–1.02)	0.89 (0.85–0.94)	0.88 (0.78–0.98)	0.91 (0.85–0.98)	
Age group							0.486
	Young adults (20–35 years)	Ref	0.96 (0.92-1.00)	0.90 (0.86–0.93)	0.88 (0.83–0.93)	0.86 (0.82–0.91)	
	Middle-aged (36–45 years)	Ref	0.94 (0.90–0.98)	0.89 (0.84–0.94)	0.90 (0.85–0.97)	0.84 (0.78–0.91)	
	Older adults (46–59 years)	Ref	0.96 (0.91–1.01)	0.91 (0.86–0.96)	0.93 (0.87–0.99)	0.92 (0.86–0.98)	
BMI Category							0.418
	Normal (< 25)	Ref	0.96 (0.90–1.02)	0.90 (0.86–0.96)	0.93 (0.87–0.99)	0.87 (0.81–0.94)	
	Overweight (25 ~ 29.9)	Ref	0.94 (0.90–0.99)	0.91 (0.87–0.94)	0.89 (0.84–0.94)	0.85 (0.81–0.90)	
	Obesity ( ≥30)	Ref	0.94 (0.90–0.98)	0.90 (0.85–0.94)	0.91 (0.86–0.97)	0.92 (0.87–0.97)	
Diabetes							0.324
	No	Ref	0.96 (0.94–0.99)	0.90 (0.88–0.93)	0.90 (0.87–0.94)	0.88 (0.84–0.91)	
	Yes	Ref	0.91 (0.85–0.99)	0.94 (0.84–1.05)	0.89 (0.80–0.99)	0.91 (0.78–1.05)	
Hypertension							0.222
	No	Ref	0.97 (0.94-1.00)	0.90 (0.87–0.93)	0.90 (0.86–0.94)	0.87 (0.83–0.91)	
	Yes	Ref	0.95 (0.90–0.99)	0.92 (0.87–0.97)	0.92 (0.85–0.99)	0.89 (0.84–0.94)	

Ratios (exp(*β*)) represent the relative change in visceral fat mass (log-transformed) compared to Q1, estimated using weighted linear regression (Model 2: adjusted for age, sex, race/ethnicity, marital status, education level, smoking, alcohol consumption, daily caloric intake, BMI, diabetes, and hypertension, as applicable)

*P*_interaction was calculated using interaction terms in the regression model

**Fig. 3:**
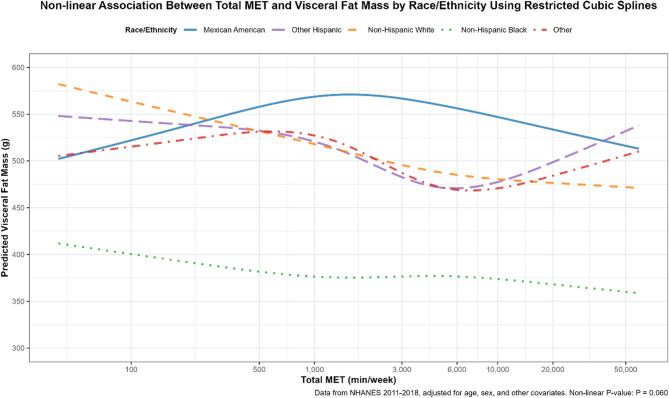
Race/ethnicity -specific association between total MET and visceral fat mass using restricted cubic splines. Note: Data from the NHANES dataset (2011–2018), including participants with total MET ≥ 40 min/week, adjusted for age, sex, marital status, education level, smoking, alcohol consumption, daily caloric intake, BMI, diabetes, And hypertension. RCS models used four knots at the 5th, 35th, 65th, And 95th percentiles of log-transformed total MET. The x-axis represents total MET (min/week), ranging from 40 to 50,000, displayed on a natural logarithmic scale (ln) with breaks at 100, 500, 1,000, 3,000, 6,000, 10,000, 20,000, And 50,000. Non-linear *P*-value: *P* = 0.060

Among racial/ethnic groups, the PA-VFM association varied significantly (*P*_interaction < 0.001). Non-Hispanic Whites, despite having the highest baseline VFM (~ 550 g at 100 MET-min/week, Fig. 3), showed a consistent decline across quintiles (Q5 vs. Q1: Ratio = 0.87, 95% CI: 0.82–0.92), as did Non-Hispanic Blacks (Q5 vs. Q1: Ratio = 0.93, 95% CI: 0.87–0.99), who had the lowest baseline VFM (~ 400 g). Other Hispanics showed a stronger VFM reduction at Q4 (Ratio = 0.84, 95% CI: 0.77–0.92), consistent with the RCS pattern of a nadir at 6,000 MET-min/week (~ 470 g), though VFM increased slightly at higher PA levels. The Other group also displayed a notable reduction (Q4: 0.88, 95% CI: 0.78–0.98). Mexican Americans, however, showed the weakest association (Q5 vs. Q1: Ratio = 0.96, 95% CI: 0.90–1.02), reflecting the inverted U-shaped RCS curve (Fig. 3).

No significant interactions were observed for age, BMI, diabetes, or hypertension on the PA-VFM relationship (*P*_interaction = 0.486, 0.418, 0.324, And 0.222, respectively). Across BMI categories, the association remained stable (e.g., Obesity Q5: Ratio = 0.92, 95% CI: 0.87–0.97). Detailed subgroup patterns are discussed further in the Discussion section.

### Non-Linear relationship

Figure 2 illustrates the sex-stratified restricted cubic spline (RCS) analysis of the association between total MET and visceral fat mass (VFM). Males exhibited a monotonic decrease in VFM with increasing physical activity (PA), suggesting a consistent benefit of higher PA levels. In contrast, females displayed a U-shaped relationship: VFM decreased from 100 to 6,000 MET-min/week (nadir ~ 410 g), then increased to ~ 450 g at 50,000 MET-min/week. The overall non-linear *P*-value was 0.418, indicating no significant non-linear association across the entire sample. However, a significant sex interaction was observed (*P*_interaction = 0.008), highlighting differential PA-VFM responses between males and females. The U-shaped curve in females suggests that moderate PA (approximately 1,680–6,000 MET-min/week, corresponding to Q3–Q4) may be optimal for reducing VFM, while extremely high PA levels (> 10,000 MET-min/week) might lead to an increase in VFM. 

Figure 3 presents the race/ethnicity-stratified RCS analysis, including five groups: Mexican American, Other Hispanic, Non-Hispanic White, Non-Hispanic Black, and Other. Non-Hispanic Whites and Non-Hispanic Blacks both showed a consistent decline in VFM with increasing PA, but their baseline levels differed significantly: Non-Hispanic Whites started with the highest VFM (~ 550 g at 100 MET-min/week), while Non-Hispanic Blacks had the lowest (~ 400 g at 100 MET-min/week). By 50,000 MET-min/week, VFM decreased to ~ 450 g for Non-Hispanic Whites and ~ 350 g for Non-Hispanic Blacks. In contrast, Other Hispanics exhibited a gradual decline in VFM, reaching a nadir of ~ 470 g at 6,000 MET-min/week, followed by a slight increase at higher PA levels. The Other group and Mexican Americans displayed more complex patterns. The Other group, starting at a similar VFM as Mexican Americans (~ 510 g at 100 MET-min/week), peaked at ~ 530 g between 500–1,000 MET-min/week, then declined to a nadir of ~ 470 g at 6,000 MET-min/week, before rising again at higher PA levels. Mexican Americans followed an inverted U-shaped curve, with VFM peaking at ~ 570 g between 1,000–3,000 MET-min/week, then declining steadily to ~ 500 g at 50,000 MET-min/week, possibly influenced by lifestyle factors (e.g., high-calorie diets) or genetic predispositions [43, 44]. The overall non-linear *P*-value was 0.060, suggesting a borderline significant non-linear association across race/ethnicity groups, with distinct PA-VFM patterns that warrant further investigation. 

### Sensitivity analyses results

Sensitivity Analyses confirmed the robustness of our primary findings. When hypercholesterolemia was added to Model 2 , the association between physical activity (PA) and visceral fat mass (VFM) remained significant, with the highest quintile (Q5 vs. Q1) showing a ratio of 0.89 (95% CI: 0.86–0.92) (Supplementary Table 1). The inclusion of hypercholesterolemia as a covariate in Model 3 was independently associated with VFM (coefficient = 0.077, *P* ≤ 0.001), reinforcing its role in the PA-VFM relationship. Using Fowler et al.’s classification [40]PA categories were distributed as follows: Inactive (0 MET-min/week, 19.4%), Low (40–499 MET-min/week, 11.5%), Moderate (500–999 MET-min/week, 9.4%), High (1,000–1,499 MET-min/week, 7.5%), and Very High (≥ 1,500 MET-min/week, 52.2%). This alternative categorization revealed a dose-response pattern consistent with the primary analysis, with the Very High group exhibiting a significantly lower VFM compared to the Inactive group (Very High vs. Inactive Ratio = 0.90, 95% CI: 0.88–0.92) (Supplementary Table 2).

## Discussion

This study, utilizing data from a large, nationally representative sample (NHANES 2011–2018), confirms a significant inverse dose-response relationship between physical activity (PA), measured in MET minutes per week, and visceral fat mass (VFM) in US adults aged 20–59. After comprehensive adjustment for demographic, Lifestyle, And health covariates, individuals in the highest quintile of PA exhibited approximately 12% lower VFM compared to those in the lowest quintile (Ratio = 0.88, 95% CI: 0.85–0.91). This finding reinforces the critical role of PA in mitigating visceral adiposity, a key risk factor for metabolic diseases such as cardiovascular disease, type 2 diabetes, and metabolic syndrome [3, 4, 6, 7].

Our results align robustly with the existing literature establishing the benefits of PA for VFM reduction. Meta-analyses, such as those by Ohkawara et al. and Recchia et al., have demonstrated a dose-response effect, with a threshold of approximately 10 MET-hours/week for significant VFM loss in obese individuals and an effect size of −0.28 for exercise-induced VFM reduction independent of caloric restriction [45, 46]. Similarly, observational studies like M Ayabe et al. also reported a negative association between PA levels and VFM [17]. Our study extends these findings by leveraging a large, diverse population and a comprehensive PA metric (MET-min/week), confirming the association across a wide range of activity levels [17].

The mechanisms underlying the PA-VFM relationship are multifaceted [47]. PA increases energy expenditure and enhances β-adrenergic sensitivity, promoting VFM loss [48]. It also improves insulin sensitivity [49]reduces inflammation [50]and modulates adipokine secretion, counteracting VFM accumulation driven by insulin resistance [51]. The U-shaped pattern in females may involve hormonal influences (e.g., estrogen, cortisol) [52–54]while racial/ethnic differences could reflect genetic predispositions or lifestyle interactions [55]. These mechanisms require further exploration, particularly at extreme PA levels.

A key contribution of this study is the identification of non-linear patterns and subgroup differences using restricted cubic spline (RCS) and interaction analyses. Significant modifications were observed by sex (*P*_interaction = 0.008) and race/ethnicity (*P*_interaction < 0.001). Males exhibited a monotonic decrease in VFM with increasing PA up to 50,000 MET-min/week, while females showed a U-shaped relationship, with VFM decreasing to a nadir (~ 410 g) at approximately 6,000 MET-min/week before increasing slightly at higher levels (~ 450 g at 50,000 MET-min/week). This U-shaped pattern, supported by a significant sex interaction, aligns with findings from Gonzalo-Encabo et al., who reported greater VFM reduction with 225 versus 300 min/week of aerobic exercise [56]. The plateau or slight increase at very high PA levels in females may reflect stress-related hormonal changes (e.g., elevated cortisol) or compensatory energy intake [52, 53]warranting further investigation into sex-specific PA thresholds. This suggests that public health guidelines might recommend moderate PA levels (e.g., ~ 5,000–7,000 MET-min/week) for women to optimize VFM reduction while minimizing potential adverse effects.

Race/ethnicity-specific analyses revealed distinct PA-VFM patterns, with significant modifications (*P*_interaction < 0.001). Non-Hispanic Whites and Non-Hispanic Blacks showed a consistent VFM decline with increasing PA, with baseline differences (~ 550 g vs. ~400 g at 100 MET-min/week), possibly due to genetic or metabolic variations, as supported by Vásquez et al., who identified ethnic differences in physical activity and body composition in NHANES data [57]and Li and Qi, who highlighted gene-environment interactions influencing fat distribution [58]. Other Hispanics And the Other group exhibited nadirs at 6,000 MET-min/week (~ 470 g), with slight increases at higher levels, while Mexican Americans displayed An inverted U-shaped curve, peaking at 1,000–3,000 MET-min/week (~ 570 g) before declining, with a borderline significant non-linear P-value (*P* = 0.060). These patterns align with findings by Karnes et al., who reported racial/ethnic variations in body fat distribution [59]and Martos-Moreno et al., who noted ethnicity-driven differences in fat distribution and metabolic responses in obesity [60]. The weaker association in Mexican Americans (Q5 vs. Q1 Ratio = 0.96, 95% CI: 0.90–1.02) may reflect higher baseline VFM, potentially influenced by dietary habits, as Yoshida et al. found a link between lower diet quality and central obesity in Mexican Americans [61]and Alemán et al., who reviewed obesity prevalence and contributing lifestyle factors among Latinx populations [62]. These findings suggest that ethnic differences in fat distribution and metabolic responses may be shaped by genetic, dietary, and socioeconomic factors, highlighting the need for culturally tailored PA interventions, such as combining PA with dietary support for Mexican Americans to enhance VFM reduction efficacy.

Subgroup analyses revealed no significant interactions for age, BMI, diabetes, or hypertension on the PA-VFM relationship (*P*_interaction = 0.486, 0.418, 0.324, And 0.222, respectively). Across age groups, the lack of interaction may seem counterintuitive, as older adults (46–59 years) exhibited higher VFM levels across PA categories compared to younger adults, possibly reflecting age-related metabolic slowing, increased inflammation, oxidative stress, or insulin resistance. However, the absence of a significant interaction suggests that PA benefits on VFM reduction may be consistent across age groups, potentially highlighting the protective role of exercise in middle-aged and older adults, consistent with findings from Keating et al. showing that objectively measured physical activity influences visceral adiposity regardless of age [63]. Similarly, the PA-VFM association was stable across BMI categories (e.g., Obesity Q5: Ratio = 0.92, 95% CI: 0.87–0.97), indicating that PA benefits are not significantly modified by BMI. However, obese individuals appeared to experience a less pronounced absolute VFM reduction, likely due to higher baseline VFM levels, which may be exacerbated by underlying factors such as insulin resistance or chronic inflammation, as supported by Lee et al. who found that obese individuals require higher PA volumes to achieve comparable VFM reductions due to metabolic inflexibility [64].

For hypertension and diabetes, the PA-VFM association was also consistent across subgroups, though notable patterns emerged. Participants without hypertension showed greater VFM reductions across PA categories compared to those with hypertension, and the latter group had fewer individuals in higher PA categories (e.g., Q5). Similarly, in the diabetes subgroup, the sample size in higher PA groups was small. These patterns may suggest that individuals with hypertension or diabetes are less likely to engage in high PA levels, possibly due to disease-related barriers or reduced exercise tolerance, This observation is supported by Duclos et al., who found that adults with chronic conditions like diabetes were less likely to meet PA guidelines, potentially due to fatigue or comorbidities [65]. Additionally, the exclusion of individuals aged 60 years and older from our sample, due to NHANES DXA scan availability and to minimize age-related confounding (e.g., sarcopenia), further reduced the number of participants in these subgroups, potentially impacting statistical power. However, the cross-sectional design precludes causal inference, and the lack of significant interaction may stem from reduced statistical power in these smaller subgroups. Future studies with larger samples and longitudinal designs are needed to explore the interplay between chronic conditions, PA engagement, and VFM dynamics.

The inclusion of hypercholesterolemia as a covariate in Model 2 strengthened the robustness of our findings, revealing its independent association with VFM (coefficient = 0.077, *P* ≤ 0.01). This aligns with established evidence linking dyslipidemia to visceral fat accumulation [66]and suggests that PA benefits may be partly mediated through improvements in lipid profiles [67, 68]. The Fowler classification supported the primary quintile-based findings, with a significant dose-response trend (*P*_trend < 0.001). However, the uneven distribution (e.g., Very High: 52.2%) and the broad Q2 range (281–1680 MET-min/week) in the quintile approach may introduce heterogeneity, potentially affecting effect size estimates. These findings underscore the need for tailored PA recommendations, particularly considering sex-specific patterns (e.g., female U-shaped association) and the trade-offs between data-driven and guideline-based categorizations.

This study possesses several strengths, notably its large sample size drawn from the nationally representative NHANES survey, enhancing the generalizability of our findings [52, 69]. We employed DXA for VFM assessment, which correlates well with gold-standard methods like MRI [53]and utilized robust statistical methods, including survey weighting and RCS modeling, allowing for the examination of non-linear dose-response relationships while adjusting for a wide range of confounders [70].

Nonetheless, limitations must be considered. The cross-sectional design precludes establishing causality; it is possible that lower VFM enables higher PA levels, or that both are influenced by other factors [71]. Our PA assessment relied on self-report (PAQ), which is subject to recall bias [72, 73]. While DXA is reliable, it may be less accurate than CT or MRI for VFM, particularly in individuals with extreme obesity [74]. Despite extensive covariate adjustment, residual confounding from unmeasured variables like specific dietary components, genetics, or sleep patterns may remain [75]. The choice of knots in RCS modeling can influence curve shape [76]and the overall test for non-linearity was not statistically significant (*P* = 0.418), suggesting the U-shape observed in women, while supported by the significant sex interaction, warrants cautious interpretation and further validation [56]. Finally, individuals with high VFM might experience chronic fatigue, potentially limiting their ability to engage in PA [77]adding complexity to the observed association.

## Conclusion

The findings confirm a significant inverse dose-response relationship between physical activity (PA) and visceral fat mass (VFM) in US adults aged 20–59, with notable differences by sex and race/ethnicity. Males exhibit a monotonic VFM decrease with increasing PA, while females show a U-shaped pattern, with optimal VFM reduction at ~ 6,000 MET-min/week, suggesting moderate PA (~ 5,000–7,000 MET-min/week) as beneficial. Race/ethnicity also influences this relationship: Non-Hispanic Whites show a consistent VFM decline, whereas Mexican Americans display a weaker association with An inverted U-shape, peaking at 1,000–3,000 MET-min/week, potentially due to dietary and genetic factors. These results underscore the importance of PA in public health strategies against obesity and metabolic disease, highlighting the need for tailored PA recommendations by sex and race/ethnicity.Future research employing longitudinal designs, objective PA measures (e.g., accelerometers), and investigations into the underlying biological mechanisms (hormonal, genetic, inflammatory pathways) is crucial to confirm causality and refine targeted intervention strategies [78–80].

## Supplementary Information

Supplementary Material 1: Supplementary Table 1. Quintile-Based Dose-Response Relationship between MET-Based Activity Groups and Log-Transformed Visceral Fat Mass

Supplementary Material 2: Supplementary Table 2: Guideline-Based Sensitivity Analysis of Dose-Response Relationship Using Fowler Classification

## Data Availability

The data supporting the results of this study are publicly available from the National Health and Nutrition Examination Survey (NHANES) database, covering the cycles from 2011 to 2018. These data can be accessed at https://wwwn.cdc.gov/nchs/nhanes/analyticguidelines.aspx#sample-design. No additional data were generated in this study beyond the analysis of the NHANES dataset.
