# Supplementary material for: Dose-response relationship between physical activity and visceral fat mass: a cross-sectional study based on NHANES 2011–2018
Source: BMC Public Health. 2025 Sep 24;25:3113. doi: 10.1186/s12889-025-24393-6 (PMC12462104; doi:10.1186/s12889-025-24393-6)
Supplement: Supplementary file 1 — Supplementary Material 1: Supplementary Table 1. Quintile-Based Dose-Response Relationship between MET-Based Activity Groups and Log-Transformed Visceral Fat Mass [file 12889_2025_24393_MOESM1_ESM.docx]

**Supplementary Table 1: Quintile-Based Dose-Response Relationship between MET-Based Activity Groups and Log-Transformed Visceral Fat Mass**

| Model | Q1 | Q2 | Q3 | Q4 | Q5 | Trend *P*-value |
| --- | --- | --- | --- | --- | --- | --- |
| Model 1 | Ref | 0.82 (0.78, 0.86) | 0.74 (0.71, 0.78) | 0.76 (0.72, 0.80) | 0.75(0.71, 0.80) | <0.001 |
| Model 2 | Ref | 0.96 (0.94, 0.99) | 0.90 (0.87, 0.92) | 0.91 (0.88, 0.93) | 0.88(0.85, 0.91) | <0.001 |
| Model 3 | Ref | 0.96 (0.94, 0.99) | 0.90 (0.88, 0.93) | 0.91 (0.88, 0.94) | 0.89(0.86, 0.92) | <0.001 |
| Ratio = exp(β), calculated using svyglm, adjusted for NHANES survey weights. Visceral fat mass (log_dxxvfatm) is log-transformed. Model 1: Unadjusted; Model 2: Adjusted for age, sex, race, marital status, education level, smoking, alcohol consumption, caloric intake, and BMI; Model 3: Further adjusted for diabetes, hypertension and hypercholesterolemia. 2. *P*_trend calculated using physical activity quintiles as a continuous variable to assess trend significance. | | | | | | |
