# Supplementary material for: Dose-response relationship between physical activity and visceral fat mass: a cross-sectional study based on NHANES 2011–2018
Source: BMC Public Health. 2025 Sep 24;25:3113. doi: 10.1186/s12889-025-24393-6 (PMC12462104; doi:10.1186/s12889-025-24393-6)
Supplement: Supplementary file 2 — Supplementary Material 2: Supplementary Table 2: Guideline-Based Sensitivity Analysis of Dose-Response Relationship Using Fowler Classification [file 12889_2025_24393_MOESM2_ESM.docx]

**Supplementary Table 2: Guideline-Based Sensitivity Analysis of Dose-Response Relationship Using Fowler Classification**

| **Model** | Inactive (0 MET-min/week) | Low (<500 MET-min/week) | Moderate (500-999 MET-min/week) | High (1000-1499 MET-min/week) | Very High (≥1500 MET-min/week) | Trend *P*-value |
| --- | --- | --- | --- | --- | --- | --- |
| **Model 1 (Sensitivity)** | Ref | 0.90 (0.86, 0.95) | 0.82 (0.77, 0.86) | 0.75 (0.70, 0.81) | 0.74 (0.71, 0.77) | <0.001 |
| **Model 2 (Sensitivity)** | Ref | 0.98 (0.95, 1.02) | 0.97 (0.93, 1.01) | 0.95 (0.91, 0.99) | 0.90 (0.87, 0.92) | <0.001 |
| **Model 3 (Sensitivity)** | Ref | 0.98 (0.95, 1.02) | 0.98 (0.94, 1.02) | 0.95 (0.91, 0.99) | 0.90 (0.88, 0.92) | <0.001 |
| Note: Sensitivity analysis using Fowler classification based on the 2018 U.S. Physical Activity Guidelines. Ratios (exp(β)) represent relative differences in visceral fat mass compared to the Inactive group. Model 1: Unadjusted; Model 2: Adjusted for age, sex, race/ethnicity, marital status, education, smoking, alcohol, caloric intake, and BMI; Model 3: Additionally adjusted for diabetes, hypertension, and hypercholesterolemia. Trend *P*-value calculated using likelihood ratio test for ordered activity levels. | | | | | | |
